# Identification of Cell-Attachment Factors Derived from Green Algal Cells Disrupted by Sonication in Fabrication of Cell Plastics

**DOI:** 10.3390/bioengineering10080893

**Published:** 2023-07-27

**Authors:** Akihito Nakanishi, Shintaro Nemoto, Naotaka Yamamoto, Kohei Iritani, Marina Watanabe

**Affiliations:** 1 School of Bioscience and Biotechnology, Tokyo University of Technology, 1404-1 Katakuramachi, Hachioji, Tokyo 192-0982, Japan; 2 Graduate School of Bionics, Tokyo University of Technology, 1404-1 Katakuramachi, Hachioji, Tokyo 192-0982, Japan; g1123026c5@edu.teu.ac.jp (S.N.); g11220402a@edu.teu.ac.jp (N.Y.); g112102759@edu.teu.ac.jp (M.W.); 3 Department of Applied Chemistry, School of Engineering, Tokyo University of Technology, 1404-1 Katakuramachi, Hachioji, Tokyo 192-0982, Japan; 4 Research Center for Advanced Lignin-Based Materials, Tokyo University of Technology, 1404-1 Katakuramachi, Hachioji, Tokyo 192-0982, Japan

**Keywords:** cell plastics, unicellular green algae, attachment factors in matrices, proteomics

## Abstract

Cell plastics which are composed of unicellular green algal cells have been proposed in previous studies. While unicellular green algae can be freely arranged using fabrication processes, a matrix is required to attach the cells together. To date, although the cell contents collected from *Chlamydomonas reinhardtii* show the possibility of attaching cells, but it is unclear which components can be considered attachment factors. Therefore, in this study, *C. reinhardtii* cells were disrupted with sonication, and the components were separated and purified with hexane. The cell plastics with only 0.5 wt% of intermediate showed similar mechanical properties to those with 17 wt% and 25 wt% of cell components that were untreated with hexane, meaning that the purified intermediates could function as matrices. The purified intermediate was composed of approximately 60 wt% of protein as the main component, and proteomic analysis was performed to survey the main proteins that remained after hexane treatment. The protein compositions of the cell content and purified intermediate were compared via proteomic analysis, revealing that the existing ratios of 532 proteins were increased in the purified intermediate rather than in the cell content. In particular, the outer structure of each of the 49 proteins—the intensity of which was increased by over 10 times—had characteristically random coil conformations, containing ratios of proline and alanine. The information could suggest a matrix of cell plastics, inspiring the possibility to endow the cell plastics with more properties and functions.

## 1. Introduction

The Sustainable Development Goals (SDGs) were adopted by the United Nations in September 2015 [1] and have led to a great deal of attention being paid to the goal of developing a sustainable society [2]. In particular, plastics—of which more than 390.7 million tons are produced worldwide and are seen as indispensable in modern society for maintaining social infrastructure and daily life—have attracted great attention regarding sustainability [3]. Highly versatile polyolefins (PO) such as polypropylene (PP) and polyethylene (PE) are used frequently in applications such as plastic bags and packaging, and they are in the limelight as recycling targets in relation to societal sustainability [4]. More than 99% of these plastics are produced from underground sources of carbon as their raw material [5], which is a highly important issue because our dependence on limited underground resources hinders the development of a sustainable society. In addition, even bioplastics, such as bio-PP, bio-PE, bio-polyester, and bio-polyimide, are poorly biodegradable in the environment [6,7], and the non-circulation of these forms of plastic waste as carbon sources is also a highly important issue in the construction of a recycling-oriented society [8]. Moreover, not only do discarded plastics remain in the environment even when they are non-circulating, but environmentally disrupted microplastics (up to a diameter of 5 mm or less) also represent a major burden to the marine environment [9]. Environmentally disrupted plastic fragments with a diameter of between 1.01 and 4.75 mm are reported to have a negative impact on marine ecosystems, especially on the food chain [9]. Therefore, to meet the demands of a sustainable and recycling-focused society, even when continuing to use plastics, the research and development (R&D) into biodegradable plastics made of non-underground resources is urgently required.

These trends have led to conducting R&D on biomass plastics that derive from biomass resources, and these studies have shown great progress thus far [10]. Biomass is eventually produced from CO_2_ as a carbon source and can be used as a resource for recyclable plastics. For instance, the R&D on biomass plastics made of lactic acid [11,12] and polyhydroxyalkanoates (PHA) [13,14] has vigorously progressed with regard to data; however, the cost issue of separating and refining the plastic resources from biomass has not been resolved to date. Moreover, although several plastics such as bio-PE, bio-PP, bio-polyester, and bio-polyimide are made of recyclable biomass, their biodegradabilities are low in the environment [8]. If biomass is to be used as a recyclable resource, it is also important to produce biomass plastics that can easily decompose in the environment. Cell plastics made of the cells of unicellular green algae have been proposed as the direct use of biomass resources without the processes of separation and purification [15,16,17,18,19]. Additionally, cell plastics are also expected to be biodegradable plastics. The green algae and biodegradable matrices are naturally degraded in the environment [20,21], and the cell plastics fabricated with their biodegradable materials will be naturally degraded in the environment [19]. In the previous report, the biodegradability of cell plastics was fabricated with starch as the matrix, showing that the cell plastics were degraded by enzymatic treatment (amylase treatment) in 24 h [17]. The cell plastics fabricated with biodegradable cells and matrices should also maintain biodegradability, such that the cell plastics are possibly able to contribute as biodegradable green plastics. Unicellular green algae such as *C. reinhardtii* have certain merits as materials: they have a rigid cell wall [22] and a constant particle size of the cells (*C. reinhardtii* strain C-9 NIES-2235: 8.4 ± 1.2 µm [19]; *C. reinhardtii* CC-125: around 4 µm [23]; and *C. reinhardtii* CC-2931 and CC-2342: around 10 µm [23]). Although there is an additional merit that the unicell can be freely placed for fabricating cell plastics [8], the unicells require the matrix to attach to each cell. Thus far, when aiming to attach each cell, polybutylene succinate (PBS) [16] and starch [17] have been used as the biomass matrices when the cell plastic is fabricated. These matrices are expected to work as attachments for cells; however, the mechanical properties of the cell plastics fabricated with these matrices, when performing a tensile test, were depressed when increasing the cell content ratios of the cell plastics. The matrices of PBS and starch can be independently filmed. These facts strongly indicate that the cells disturb the film formation of the matrices, implying no attachment between the cells and matrices. Therefore, to increase the attachment ability between the cells and matrices, the cell contents of *C. reinhardtii* were used as the matrix of the cell plastics, since *C. reinhardtii* can attach to each other in certain cases [19]. In the analyses, the cell content was sticky, and it was confirmed that they did not exist as an independent film form. Through using the cell contents, the cell plastics containing 75 wt%~ of the cells were fabricated to analyze the mechanical properties (which were assessed by a tensile test). As a result, the Young’s modulus and maximum stress were enhanced with increasing ratios of the cells; their properties were shown at the highest values when at 79 wt%, and those which were depressed increased to over 79 wt%. For the matrices that have been fabricated, their cell plastics can thus far exist as an independent film form; furthermore, their mechanical properties in a tensile test simply decreased when increasing the ratio of the cells in the cell plastics. The lower mechanical properties of cell plastics that had higher ratios of the cells, compared to those with lower ratios of the cells, could possibly be attributed to the fact that the cells were mixed in as impurities with the originally independently film-formed matrices; additionally, the non-attachment between the cells and the matrices is also implied. Although this could suggest an attachment between the matrix and the cells, what components worked as the attachment factors was also found.

This study aimed to reveal the attachment factors in the cell contents when the matrix is attached to the cells. The cell contents of *C. reinhardtii* were fractionated with hexane, and the cell plastics were fabricated with the fractionated contents as the matrices. The mechanical properties of the cell plastics were analyzed with tensile tests, and the function of the matrices was evaluated. Furthermore, the composition of the protein, carbohydrate, and lipid in the fractionated contents was evaluated. The protein in the contents was purified with hexane and was especially evaluated with proteomic analysis to identify the attachment factors. This study is the first report to suggest what attachment factors exist in cell-plastic formation. Although the cell-attaching factors have not been elucidated in previous studies on cell plastics, if the factors attaching the cells could be clarified, there is a possibility that cell plastics with improved mechanical properties could be produced when using the properties of these attachment factors. In addition, this cell–cell attachment principle could be used as a theory of attachment in the fabrication of cell plastics, which require the effective attaching of single cells.

## 2. Materials and Methods

### 2.1. Use of Cells as Resources of Cell Plastics

*Chlamydomonas reinhardtii* strain C-9: NIES-2235 was obtained from the National Institute for Environmental Studies. The strain was grown in a photo bioreactor (80 L; 25 °C; 150 µm·m^−2^·s^−1^; 15,000 ppm-CO_2_; 0.05 vvm) in a BG-11 medium [24], and the lyophilized cells were prepared by Meravi Ltd. The cells themselves were used as the resources of the cell plastics and were manipulated to prepare the matrices.

### 2.2. Supply of Matrices

**Figure 1:**
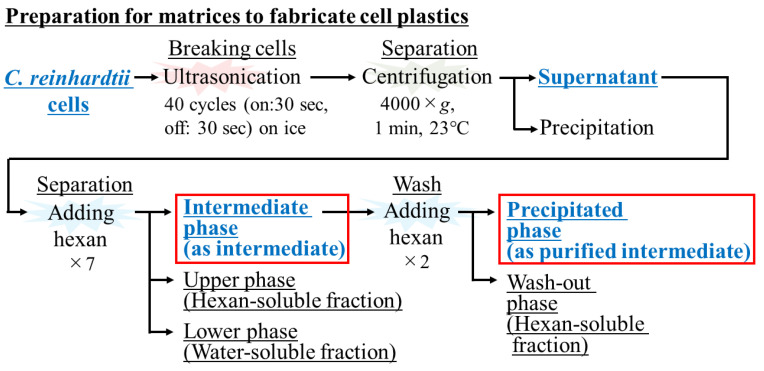
Preparation of the matrix to fabricate cell plastics. The intermediate and purified intermediate were prepared from the contents of cells that were treated by sonication, separation with centrifugation, and mixing with hexane.

The matrices for the cell plastics were recovered as cell contents, as well as the intermediate, and purified intermediate treated with hexane (Figure 1). Cell content: *C. reinhardtii* strain C-9: NIES-2235 was treated with ultrasonic homogenization and was conducted as follows: an ultrasonic homogenizer Smurt NR-50 M operating at 20 kHz with an ultrasonic horn and a 3 mm diameter tip NS-50 M-MT3 (Funabashi, Chiba, Japan) was employed. Two hundred mg of cells were suspended with 20 mL of distilled water in a 50 mL polypropylene centrifuge tube. After preparation, 1 cm of the end of the chip was inserted into the cell suspension, and the cell suspension was sonicated (on: 30 s; off: 30 s, 40 cycles) on ice. The sonicated liquid was then centrifuged (2500× *g*, 23 °C, 3 min) in order to be separated into a liquid phase and a precipitate phase, and the liquid phase was collected. The recovered liquid was again centrifugated (2500× *g*, 23 °C, 3 min), and the liquid phase was recovered as cell contents. Intermediate: 20 mL of hexane was added to the collected cell contents and thoroughly stirred. The suspension was then centrifuged (2500× *g*, 23 °C, 3 min) and fractionated into an upper phase (organic phase), an intermediate phase, and a lower phase (water phase). The middle phase was scraped off with a spatula seven times and collected. Purified intermediate: the intermediate collected in a Petri dish was mixed with 20 mL of hexane, and the liquid phase was discarded. This operation was repeated twice to purify the intermediate, and the insoluble residue was collected as purified intermediate.

### 2.3. Fabrication of the Cell Plastics

The cell plastics were prepared with *C. reinhardtii* C-9: NIES-2235 cells and matrices (cell content; intermediate; purified intermediate). First, the cells were washed twice as follows: 200 mg of dried cells were soaked into deionized water (DW), centrifuged (2500× *g*, 23 °C, 3 min), and collected. Next, the cells were collected in 1 mL of DW and transferred to a molding vessel (7.0 cm diameter × 2.5 cm depth, Daiso Industries Co., Ltd., Hiroshima, Japan); the intermediate and purified intermediate were also added to the molding vessel and were then mixed by pipetting. The cell matrix mixtures were dried in a thermo-hygrostat chamber THR040FB (Advantec, Tokyo, Japan) at 25 °C and 80 RH% humidity to fabricate the cell plastics.

### 2.4. Tensile Test

The mechanical properties of the Young’s modulus, maximum stress, and strain of the cell plastics were evaluated with a tensile strength tester INSTRON 3342 (INSTRON, Norwood, MA, USA) [15,16,17,18,19,25]. The cell plastics were cut into rectangles. The maintained crosshead rate was 1.00 mm·min^−1^. A load–displacement curve was plotted by analyzing the test samples. To calculate the Young’s modulus, maximum stress, and strain, a stress–strain (S–S) curve was plotted by dividing the load and displacement by the cross-section and the initial length of the test samples, respectively. The thickness of the cell plastics was measured by the SEM observations that were oriented toward the cross-sections of the test samples. The Young’s modulus was obtained with the slope of an initial straight line, which was approximated via the least-squares method. The maximum stress was defined as the maximum value of stress in a tensile test. The strain was expressed as when the elongation value broke, showing as 100× (broken length/original length). The Young’s modulus, maximum stress, and strain of each film were the averages of the three test samples, and these were obtained by preparing three different films.

### 2.5. Evaluation of Composition in Matrix

The weights of the carbohydrates, lipids, proteins, and ashes were obtained, and those contents were calculated with the weights of the matrices in weight percent (wt%) to evaluate the compositions in the matrices. Evaluation of carbohydrate quantity: the carbohydrate in the matrices was quantified by the anthrone method [26,27]. A matrix was added to 0.004% (*w*/*v*) anthrone in 75% H_2_SO_4_ on ice and stirred well. The mixture was then placed in a block incubator at 100 °C for 15 min. After 10 min on ice, the mixture was stirred well via vortexing. The correlation between absorbance at 620 nm and carbohydrate content was indicated by a calibration curve that was prepared with glucose, and the reaction supernatant was quantitatively evaluated with the calibration curve after a 20-fold dilution. Evaluation of lipid composition and quantity: the cells were broken with 0.5 mm glass beads, and then the total extracted lipids from the intermediate and purified intermediate were methyl esterified with a fatty acid methylation kit (Nacalai Tesque, Kyoto, Japan) [28,29]. The fatty acid methyl esters were identified and quantified with a capillary gas chromatograph GC-2025 (Shimadzu, Kyoto, Japan), which was equipped with a DB-23 capillary column (60 m, 0.25 mm internal diameter, 0.15 μm film thickness) (Agilent Technologies, Santa Clara, CA, USA) and was followed by the previous method [29]. Heptadecanoic acid (Sigma-Aldrich Co., St. Louis, MO, USA) was used as an internal standard, and rapeseed oil (Merck KGaA, Darmstadt, Germany) was used as a quantitative standard. Evaluation of protein quantity: the protein in the matrices was quantified with a bicinchoninic acid protein assay. The total protein content was determined using a bicinchoninic acid protein assay kit (Takara Bio, Shiga, Japan). Evaluation of ash: Matrix was lyophilized with a Refrigerated CentriVap Centrifugal Vacuum Concentrator (−4 °C, 24 h) (Labconco Corporation, Kansas City, MO, USA). The lyophilized matrix was moved to thermostable vessels and the weights were indicated with a HR-202i (A&D company, Limited, Tokyo, Japan). The matrix in the thermostable vessel was placed in an electric furnace of VTDS-64K (Isuzu Manufacturing Co., Ltd., Niigata, Japan) at 525 °C for 5 h. The ash weight was then determined gravimetrically.

### 2.6. Observation by Microscopy

The cell plastics were observed with an optical microscope BX53 equipped with an objective lens ((100×): UPLSAPO100XO, fluorescent mirror unit: T01-4214-23 U-FGW (excitation filter: 530–550 nm; absorption filter: 575 nm IF)) and a digital camera (DP74 Digital camera: DP74).

### 2.7. Evaluation of Remained Cell Numbers as Structures of Cell Plastics

The cell numbers used for the fabrication of the cell plastics were evaluated with a standard curve of dry cell weights (DCWs) versus those numbers. On the other hand, the cell numbers remaining in the cell plastics were suspended with DW and counted with a hemocytometer. The numbers of cells remaining in the cell plastic were divided by the numbers of cells used to fabricate the cell plastics and then expressed as a percentage.

### 2.8. Analysis of Proteomics

The *C. reinhardtii* cell contents and purified intermediates were prepared at 10 mg for the purpose of performing the proteomic analysis (Kazusa DNA Res. Inst., Chiba, Japan). The samples were mixed with 100 mM of Tris buffer (pH = 8.0) adjusted to 4% SDS and 20 mM of NaCl, and the protein was dissolved by a sealed ultrasonic disruption machine. The protein concentration was evaluated with a BCA assay and adjusted to a protein concentration of 0.5 μg·μL^−1^ with a 100 mM of Tris buffer (pH = 8.0) containing 4% SDS and 20 mM of NaCl. To cleave the S–S bond of the protein, TCEP containing 20 μg of protein was added to the solution to a final concentration of 20 mM, and the mixture was incubated at 80 °C for 10 min. Iodoacetamide (IAA) was added to alkylate the cysteine residues and was adjusted to 30 mM as a final concentration. The solution was incubated at 23 °C under a light shield for 30 min. The Sera-Mag Speed Bead Carboxylate-Modified Magnetic Particles (Hydrophobic) and Sera-Mag Carboxylate-Modified Magnetic Particles (Hydrophobic) were provided by Cytiva and were mixed at a 1:1 (*v*/*v*) ratio. The SP3 beads were prepared after three times wash with DW and were finally adjusted to 8 μg·μL^−1^ with the DW. The alkylated samples were mixed at 23 °C for 20 min after the addition of 20 µL of SP3 beads and an additional three times of the liquid volume of ethanol. Then, the beads were washed twice with 80% ethanol, and 100 μL of 50 mM Tris-HCL pH 8.0 was added for mixing. Trypsin/Lys-C Mix (Promega) 500 ng was added to digest the protein into the peptide fragments, and the mixture was incubated at 37 °C overnight. The samples were processed in a sample-sealing sonicator after adding 20 μL of 5% TFA, desalted in a reversed-phase spin column (GL-Tip SDB, GL Sciences), and dried in a centrifugal evaporator. Then, 2% ACN-0.1% TFA was added into the samples and adjusted to 100 ng·μL^−1^ to dissolve the peptides with a sealed ultra-sonicator. Those samples were analyzed with an UltiMate 3000 RSLCnano LC System equipped with a Q Exactive HF-X (Thermo Fisher Scientific) that was operated under its ESI positive mode. The analysis followed the below conditions and amount of injected peptides: 100 ng; column: 75 µm (inner diameter) × 120 mm (length) (Nikkyo Technos, Co., Ltd., Tokyo, Japan); column temperature: 40℃; mobile phase condition: phase-A distilled water containing 0.1% formic acid and phase-B CAN containing 0.1% formic acid; and gradient condition of mobile phase: 7% of phase-B up to 35% (0–22 min), 35% of phase-B up to 65% (22–28 min), 65% of phase-B (28–30 min). The raw data were searched against in silico predicted spectral library using DIA-NN (version:1.8.1, https://github.com/vdemichev/DiaNN accessed on 28 November 2022). First, the in silico-predicted spectral library was generated from *C. reinhardtii* UniProtKB/SwissProt database (Proteome ID UP000006906, 18,829 entries) using DIA-NN. The DIA-NN search parameters were as follows: protease, trypsin; missed cleavages, 1; peptide length range, 7–45; precursor charge range, 2–4; precursor mass range, 495–745; fragment ion *m*/*z* range, 200–1800; mass accuracy, 10 ppm; static modification, cysteine carbamidomethylation; enabled “Heuristic protein interferences”, “Use isotopologues”, “MBR”, and “No shared spectra”. Additional commands were set as follows: “mass acc cal 10”, “peak translation”, and “matrix spec q”. The protein identification threshold was set at <1% for both peptide and protein false discovery ratios (FDRs). The normalization parameter “Cross-run normalization” was set at “RT-dependent”. The statistical calculation was performed by Perseus v1.6.15.0. The protein intensities were transformed to log2 and filtered for each group; at least one group contained a minimum of 70% valid values. The missing values of proteins were imputed by random numbers based on a normal distribution (width parameter = 0.3, downshift parameter = 1.8). The fold change and content ratio of the amino acids are shown below. Fold change: the values of the averaged protein intensity in the intermediate after hexane purification/averaged protein intensity in the cell contents without hexane purification; content ratio of amino acids (%): 100 × (each AA residue number of exposed protein parts/all AA residue numbers of the exposed protein parts). The images of the three-dimensional models of proteins, accession numbers, and ORF name were obtained from the UniProt database. Fold changes are shown as the values of the ‘averaged protein-intensity in intermediate after hexane-purification/averaged protein intensity in cell contents without hexane-purification’.

## 3. Results

### 3.1. Evaluation of the Composition of Cells Prepared by Sonication

**Figure 2:**
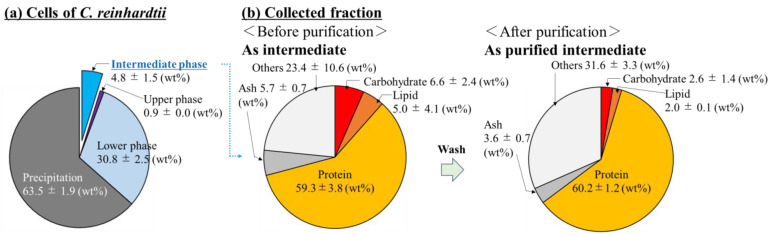
Collection ratio of the cell contents and composition ratios of the intermediate and purified intermediate. Composition ratios of the (**a**) cells (**b**) collected phases before/after hexane purification were evaluated with carbohydrates, lipids, proteins, ashes, and others. Both collected phases were derived from the intermediate phase of cells, and both were defined as intermediate and purified intermediate before/after hexane purification. The averaged value and standard deviation (SD) were shown by six-replicated experiments (*n* = 6).

After sonication to the cells, the ratio, as the wt% of the supernatant and precipitate, were determined; furthermore, the composition of the intermediate, upper (organic), and lower (aqueous) phases were also revealed in terms of wt% when the cell contents were fractionated with hexane (Figure 2a). As a result, the cell content was approximately 36.5 wt%; the contents of the recovered cell that were fractionated with hexane were recovered at 4.8 ± 1.5 wt% in the intermediate phase, 0.9 ± 0.0 wt% in the upper phase, and 30.8 ± 2.5 wt% in the lower phase. The composition (carbohydrates, lipids, proteins, ashes and others) in the intermediate and the additionally purified intermediate were shown in the same manner as the wt% (Figure 2b). The results showed that the intermediate was occupied by 59.3 ± 3.8 wt% of protein as the highest composition, 6.6 ± 2.4 wt% of carbohydrates, 5.0 ± 4.1 wt% of lipids, 5.7 ± 0.7 wt% of ashes, and 23.4 ± 10.6 wt% of other. On the other hand, the intermediate purified with hexane was also shown as 60.2 ± 1.2 wt% of protein as the highest composition, 2.6 ± 1.4 wt% of carbohydrates, and 2.0 ± 0.1 wt% of lipids, 3.6 ± 0.7 wt% of ashes, and 31.6 ± 3.3 wt% of other. Regarding the other aspect, the composition of the component expressed as others increased from 23.4 wt% to 31.6 wt% by purifying the intermediate phase with hexane.

### 3.2. Tensile Test of Cell Plastics Composed of Intermediate as a Matrix

**Figure 3:**
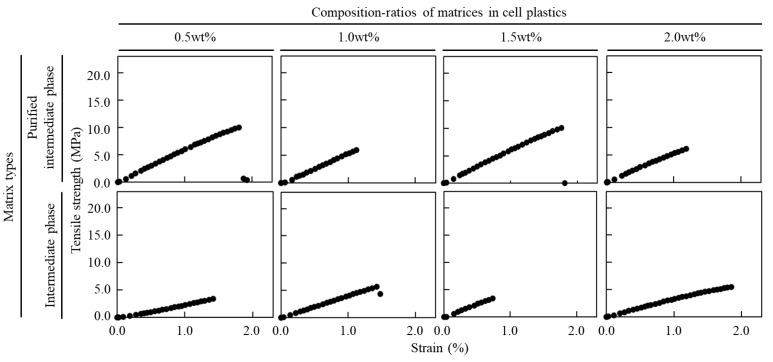
The strain–stress curve of cell plastics with intermediate and purified intermediate in a tensile test. Cell plastics contained 0.5 wt%~2.0 wt% of intermediate, and the purified intermediate was fabricated. The data from the tensile tests are plotted as an S–S curve.

All these phases were collected from an organic phase, an intermediate phase, and an aqueous phase by the addition of hexane that could not be made into films. Each phase was mixed with cells as the matrix and the mixture was then prepared to fabricate the cell plastics, resulting in the mixture that only had an intermediate as the matrix being formed into cell plastics. Then, the cell plastics with different content ratios of intermediates were prepared, and those mechanical properties were analyzed in a tensile test (Table 1). The results showed that cell plastics containing only 0.5~2.0 wt% intermediate exhibited 227.3~422.1 MPa of Young’s modulus, 3.5~4.6 MPa of maximum stress, and 1.1~1.6% of strain. In detail, the maximum stress and strain showed no correlation with the content ratios of the intermediates; however, the Young’s modulus tended to reach its maximum value when the ratio of the intermediate was 1.5 wt%. The intermediate was additionally washed out with hexane, and the purified intermediate was collected. The purified intermediate was powdered and did not exist as an independent film. However, the powder of the purified intermediate could work as the matrix, meaning that the cell plastics were fabricated with them. The mechanical properties of these cell plastics were also analyzed by tensile test (Table 1), and it was shown that those cell plastics with only 0.5~2.0 wt% of the content ratios of the purified intermediates exhibited 550.1~564.4 MPa of Young’s modulus, 5.1~7.7 MPa of maximum stress, and 0.4~1.4% of strain. By using the purified intermediate as the matrix, although the strain of the cell plastics remained almost the same or tended to decrease slightly, the Young’s modulus and maximum stress tended to increase. The purified intermediates were used as the matrices and, as they were deeply related to the values of Young’s modulus, they tended not to scatter. On the other hand, the content ratios of the purified intermediate did not show a clear correlation with the values of the mechanical properties observed in the tensile test. To additionally evaluate how the mechanical properties of each cell plastic were reflected by the refining of the intermediate and content ratios of them as a matrix, the S–S curves obtained from the tensile test were analyzed (Figure 3). All the cell plastics exhibited an elastic range, and all failed at once after reaching the maximum stress value.

### 3.3. Evaluation of Cells as the Structural Components of Cell Plastics

**Figure 4:**
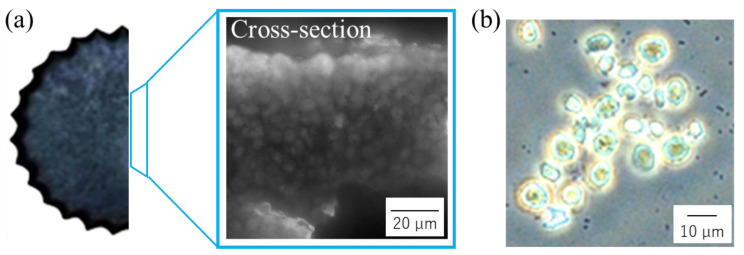
Images of optical microscopy. Cell plastics containing 0.5 wt% of purified intermediate were observed with optical microscopy. Scale bars are 20 μm and 10 μm in a (**a**) cross-section (**b**) cells after re-suspension with DW.

To show the presence of the cells that were used as structural components of cell plastics and dispersion rather than localization on the surface, the cross-section surface of the cell plastics with a purified intermediate as the matrix was observed with an optical microscope (Figure 4a). Image analysis revealed numerous cell-sized particles on the surface of the cross-section of the cell plastics. Regarding the process of fabricating the cell plastics with the purified intermediate as the matrix, the cell plastics were again carefully suspended in DW and observed under an optical microscope to determine whether the cells remained as a structure without autolysis or other such degradation (Figure 4b). As a result, numerous cell-like structures, approximately 8~10 µm in size, were detected, and 78.53 ± 19.21% of the remaining cells in the cell plastics were confirmed.

### 3.4. Suggestion of Attachment Factors to Cells in Cell Plastics

**Figure 5:**
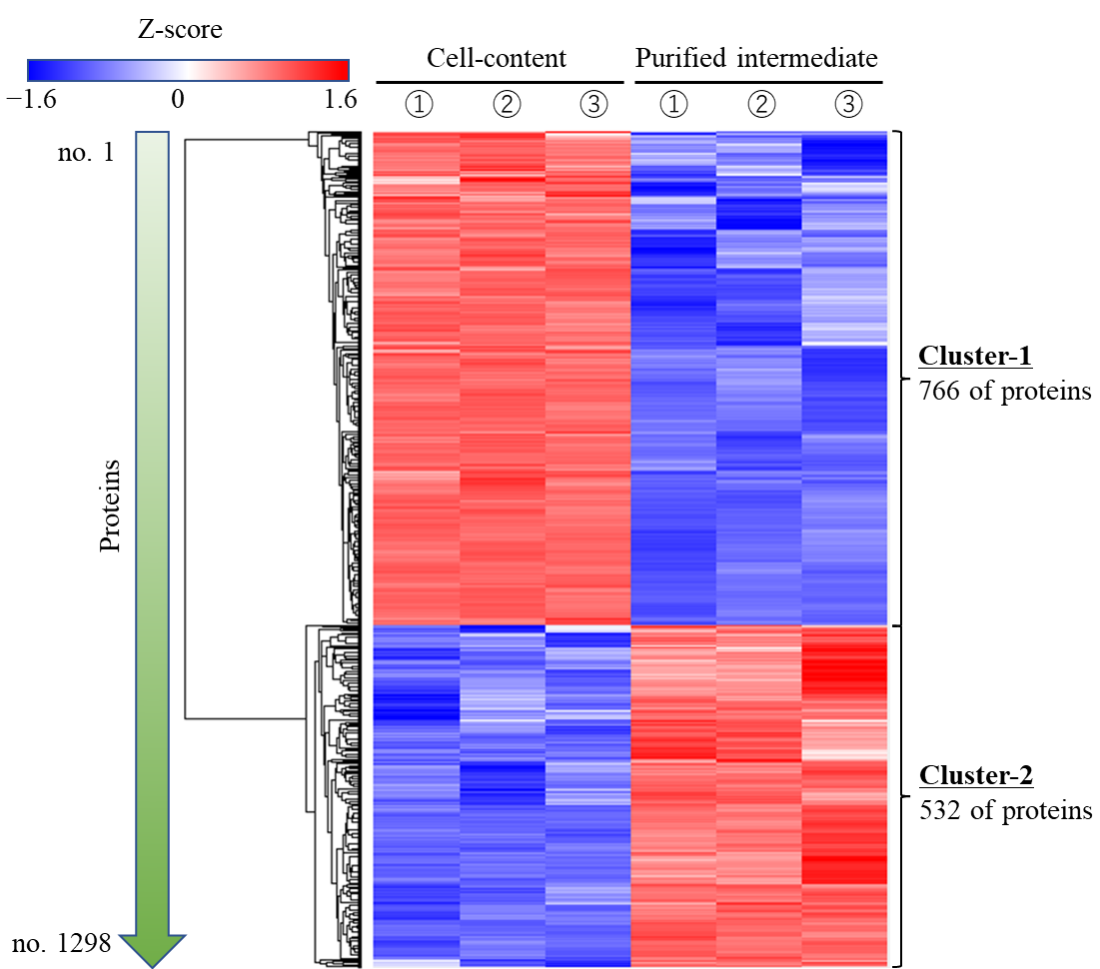
Heatmap based on the Z-score of all proteins detected by proteomic analyses. ①–③ indicated replicated trials. The proteins in the cell contents and purified intermediates were evaluated via proteomic analyses. The quantitative values for each protein were converted to log_2_ values and are shown as protein intensities. The Z-scores are gradually colored from blue (−1.6) to red (1.6). The proteins depressed/enhanced with hexane treatment are clustered in Cluster 1 and Cluster 2, respectively. The tree diagram on the left side of the heatmap shows the results regarding the similarity of expression profiles for each protein. Proteins with similar tendencies in terms of increasing or decreasing in expression are placed closer together.

**Figure 6:**
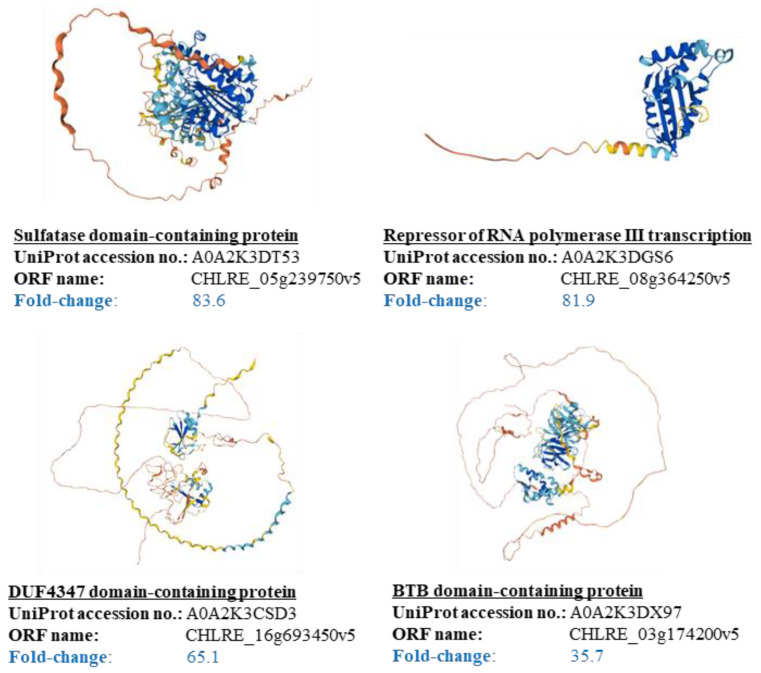
Three-dimensional models of the proteins that had a high occurrence in the purified intermediate. The images for the three-dimensional models of the proteins, accession numbers, and ORF name were obtained from the UniProt database. Fold changes are shown as the values of the ‘averaged protein intensity in intermediate after hexane-purification/averaged protein-intensity in cell contents without hexane-purification’.

These results suggest that the purified intermediate could work as an attachment factor to the cells. However, what factors could function as attachment factors in cell plastics has not yet been clarified. Therefore, an omics analysis was performed on the protein that constituted approximately 60 wt% of the purified intermediate, and the results were evaluated by comparing it with those of the same analysis on the hexane-untreated contents. First, a total of 1298 proteins were identified and the logarithmic protein intensities were determined for each protein to clearly indicate their abundance (the identified proteins: 5894 in intermediate; 5800 in the purified intermediate), resulting in all protein intensities being heat-mapped via the Z-score (Figure 5). The Z-score of each protein could be clustered almost without any positive or negative differences even after repeated trials, and the purified intermediate was divided into a cluster containing 766 specifically reduced proteins (Cluster No. 1) and a cluster containing 532 specifically increased proteins (Cluster No. 2). Subsequently, 49 proteins grouped in Cluster No. 2—which were 10 times larger than the protein intensities of the purified intermediate that were divided by those of the cell content—were identified, indicating that they were largely present and remained specifically in the purified intermediate (Supplemental Table S1). The structures of those proteins that were largely present and specifically remaining in the purified intermediate were evaluated (Figure 6). The comparison of higher-order structures of those proteins in the protein database UniProt revealed specific α-helix and linear structures that were present in long exposures outside of the proteins. The amino acid frequencies of the outer structures of the proteins that occur with high frequency in the purified intermediate (Table 2) were analyzed. Specifically, the Pro_397_ to Ser_510_ of the sulfatase N-terminal domain-containing protein as the outer region, and Ser_198_ to Val_235_ of the repressor of RNA polymerase III transcription as the outer region corresponded to this fact. The amino acid composition of these sequences was remarkably high in proline and alanine. For example, in the sulfatase N-terminal domain-containing protein, the 40 residues between Pro_411_ and Pro_450_ were exposed as outside of the protein and were 50% proline.

## 4. Discussion

The composition of the aqueous phase after sonication was evaluated (Figure 2). In this study, the cell sonication conditions were 20 kHz in 20 mL of DW. On the other hand, Wang et al. treated two species of microalgae *Scenedesmus dimorphus* and *Nannochloropsis oculata* with a high-frequency (3.2 MHz) focused ultrasound and with a low-frequency (20 kHz) non-focused ultrasound [30]. The authors found that focused ultrasound effectively destroyed the microalgal cells, and the effectiveness of cell disruption depended on the targeted cells. In addition, ZhiPeng et al. reported that more than 80% of *Chlamydomonas reinhardtii* cells were disrupted with a low-frequency ultrasound (35 kHz) after a 5 min treatment [31]. Based on these previous studies, although our cell disruption process might not have been optimized, the recovery ratio of approximately 36.5 wt% of cell contents was not unnatural and was enough for our research. Generally, in the scenario that the cell contents are mixed with aqueous and organic solvents, the intermediate phase contains either structurally disentangled proteins or amphiphilic molecules such as lipids. In this study, the intermediate phase was gelatinized by the addition of hexane, and its morphology indicated that the intermediate could contain not only amphiphilic components, but also certain organic and water-soluble components. The cells assumedly contained roughly 20 wt% of protein in this study since the cells used for fabrication of the cell plastics were before nitrogen deprivation took place [15]; however, the actual wt% of the recovered protein was 2.8 wt% (4.8 × 0.593 wt%), meaning that the protein recovery was less than 20% and not high. Therefore, the recovery was not for all proteins but for the partial ones that had the hexane treatment. However, once the proteins were recovered with hexane, their proportions hardly changed after purification, suggesting that this extraction and purification method could present a few problems for protein purification.

The cell plastics were prepared by varying the content of the intermediate, and the mechanical properties decreased after the optimum point at the content of 1.5 wt%, suggesting that the cells were possibly attached to each other with the matrix (Table 1). In addition, the mechanical properties of the cell plastics were prepared with approximately only 1.5 wt% of the intermediate, and these were close to those with 17 wt% and 25 wt% as the matrices [19]—thus suggesting that the cell attachment factors could be contained in the intermediate. However, the standard deviations were still scattered widely, such that the presence of impurities was suggested, indicating that the further purification of the intermediate was required to elucidate the attachment factor. According to the data shown in Table 1, the cell plastics that were fabricated even with 0.5 wt% of the purified intermediate exhibited a higher Young’s modulus and maximum stress than those with the hexane-untreated intermediate. Additionally, the small variation in standard deviation of Young’s modulus suggests that the impurities were possibly removed and that the attachment factors were purified. Purification of the intermediate with hexane did not improve the strain property, indicating that the material broke without showing ductility. This tendency is easily observed in glass and ceramics [32,33], and cell plastics are also brittle. This might be due to the lack of elasticity in the cells and the purified intermediate as the matrix, which could affect the mechanical properties of the cell plastics. These properties are similar to the mechanical properties of cell plastics that are fabricated with hexane-untreated cell contents as the matrices [19]. In addition, the comparison could suggest that the attachment factors in the cell contents may have been purified and used in this study. Following the tensile tests, the materials could be broadly divided into brittle materials, which ruptured outside the elastic region, and ductile materials, which remained in the plastic region after leaving the elastic region [33,34,35]. As shown in Figure 3, the cell plastic ruptured at once after experiencing the maximum stress and did not show a plastic zone, indicating that it was a brittle material with mechanical properties and has only an elastic deformation, such as in glass and ceramics [32,33], rather than a metallic material with a plastic region [33]. As with the evaluation of the strain shown in Table 1, the cell plastics were similar to brittle materials, as they were brittle like glass and ceramics. Since previous cell plastics that were fabricated by the hexane-untreated cell contents as matrices broke at once after showing the maximum stress values [19], the cell plastics in this study also showed similar mechanical properties. Therefore, it is possible that the attachment factors involved in the cell contents were also recovered and were used in the fabrication of the cell plastics.

The cell plastic images, previously taken by scanning electron microscopy (SEM), primarily showed that cell-like structures existed on the surface of the cell plastics [19]; however, what was inside the cell plastics was constructed with dispersed cells as the structural components were not displayed. The previous cell plastics were fabricated directly with the cell contents as the matrices contained over 10 wt% of the contents, such that those cells resulted in cells that were masked with the matrices; moreover, these cells were observed with difficulty in the SEM images. The cell aggregation in the cell plastics made of cells and no attachment matrices induced a degradation and instability of the mechanical properties because the cells that do not to attach each other cause stress concentrations [33]. For example, for the cell plastics that were prepared using PBS and starch as the matrices, the cells that attached to the matrices were investigated based on the evaluation of the mechanical properties that were assessed in the tensile tests [16,17]. The cells that were dislodged from the matrices as the main components in the cell plastics might exist as a clump inside and may not fit to the matrices without dispersing. However, the optical microscope image of the cross-section shown in Figure 4a did not show any significant localization of cells and differed significantly from the previous cases. Composites with homogeneously dispersed particles are known to have higher load-bearing properties [36]. In this study, the analysis of optical microscopy images could reinforce the possibility that the matrix attachment to cells could be suggested by analyzing the mechanical properties. The cell-like structures in the cell plastics were confirmed by optical microscopy, as shown in Figure 4b, indicating that the cell plastics were composed of cells. Furthermore, 78.53 ± 19.21% of the high ratio of the cell recovery strongly indicates that the cells still function as components of the cell plastics even after structural formation, and that these cells have escaped significant damage.

Proteomic analyses of *C. reinhardtii* have been performed on various targets, such as in the study of Stauber et al. for light-harvesting proteins [37], and by Diniz et al. for flagella [38]. On the other hand, however, this study is the first proteomic analysis that is targeted toward the remaining proteins after treatment with hexane (Figure 5). According to the database on UniProt (15 May 2023), 14,582 of the proteins in *C. reinhardtii* were set as ‘Predicted’, and there were 1298 proteins that were collected under the processes of sonication and hexane treatment, meaning that 8~9% of the total protein species were recovered in this study. Although not all proteins are always expressed, the fact that approximately only 2.8 wt% of proteins were recovered from the prepared cells suggests that they had a low recovery ratio of less than 10%, thus suggesting that a diverse number of proteins might have occurred due to the recovery method. The proteins detected in the purification intermediate were the nuclear-localized repressor of RNA polymerase III transcription (fold change: 81.9); the nuclear-localized UBC core domain-containing protein (fold change: 81.5); the protein kinase domain-containing protein localized to actin filaments (fold change: 41.7); and the C3H1-type domain-containing protein localized to cytoplasm (fold change: 35.7)—suggesting that the cells could be disrupted by the methods of sonication and hexane treatment. What was particularly interesting was that almost all the proteins could be clustered without any positive or negative difference in Z-score, even after repeated attempts. This indicates that the hexane purification worked reproducibly for the purification of these specific proteins, and this had a great impact on the results of the analysis.

The results of the proteomic analysis in this study showed that random coil conformations consisting of proline-rich regions (PRRs) were characteristically respected in the outer structures of the proteins with high fold changes (Table 2). In general, PRRs are known to have difficulty maintaining a higher-order structure and tend to be linear because of their inability to form hydrogen bonds between peptide bonds. Specifically, the bulkiness of the side chain of proline, known as the α-helix breaker or β-sheet breaker [39], makes it difficult to maintain a higher-order structure. These PRRs are also known to maintain the structures of cells and tissues by acting similar to a string; they also bind non-stoichiometrically rather than functioning alone [39], and they significantly contribute with acting to keep peptidoglycan phases together [40]. For instance, proline-rich proteins have long been known to bind and adhere as membrane-bound coat proteins to compounds such as the procyclin of Trypanosoma brucei [41], the M protein in Group C of *Streptococcus equi* [42], the IgA receptor in Group B of Streptococcus sp. [43,44,45] as peptidoglycan-binding proteins, and the p70 pertactin of *Bordetella parapertussis* [46] as an outer-membrane protein involved in cell adhesion. Furthermore, *C. reinhardtii* has extracellular matrix proteins that are similar to hydroxyproline-rich glycoproteins, which contribute to cell–cell junctions and are homologous to the sex pheromones in its cell wall [31,47]. Although there is a possibility that the collected proteins in this study were denatured by this hexane treatment, Miyake et al. reported that no denaturation occurred when bacteriorhodopsin was suspended in hexane [48], meaning that the purified intermediates could be recovered without denaturation. In addition, lower-order structures of the proteins such as random coil conformation could possibly contribute to the attachment to cells rather than their higher-order structures. Therefore, there is a possibility that the random coil conformation in the purified intermediates was maintained even after the hexane treatment, and that it deeply contributed to the attachment factors between the cells. Proline residues are generally abundant in the proteins formed with random coil conformation, such as collagen [49] and elastin [50], and proline is known to be deeply involved in protein–protein interactions. In view of the above, it is expected that the findings in this study will possibly be used, in the future, for matrix selection to create cell plastics with diverse properties.

## 5. Conclusions

In this study, the identification of the attachment factors of cells in cell contents was attempted. The cell contents were previously examined to assess the possibility of attaching the cells together as a matrix, and the intermediate that was fractionated from the cell contents with hexane was shown to serve as the matrix for the cell plastics in this study. The function of the purified intermediate as a matrix was particularly distinctive, and the cell plastics containing only 0.5 wt% purified intermediate exhibited similar mechanical properties to those of the cell plastics containing 17 or 25 wt% of the non-treated cellular contents. Content analysis for the purified intermediate revealed that nearly 60 wt% of it was protein, and the proteomic analysis implied that most of the purified proteins had lower-order structures with a high content ratio of proline and alanine in their outer arms. This finding suggests the possibility that characteristic protein structures with α-helices and linear structures in the outer shell could be factors that could be used to attach the cells together in the cell plastics. These results indicate that proteins with typical structures such as collagen and elastin might effectively work in the formation of cell plastics. This discovery results in the proposal of a matrix for the attachment of cells, one that is based on the structure of this protein, and which also shows the possibility of fabricating the cell plastics with various mechanical properties by diversifying the indirect attaching regions to the cells.

## Figures and Tables

**Table 1:** Mechanical properties of the cell plastics fabricated with intermediates with/without hexane purification as the matrices in a tensile test. Values are the averages of three-replicated experiments ± SD.

Hexane Purification	Matrix Contents (wt%)	Young’s Modulus (MPa)	Tensile Strength (MPa)	Strains (%)
w/	0.5	227.3 ± 29.5	4.6 ± 2.0	1.6 ± 0.4
	1.0	358.3 ± 143.2	3.6 ± 1.0	1.3 ± 0.4
	1.5	422.1 ± 105.9	4.3 ± 2.6	1.1 ± 0.3
	2.0	332.5 ± 110.0	3.5 ± 1.8	1.2 ± 0.6
w/o	0.5	562.9 ± 11.8	7.7 ± 3.3	1.3 ± 0.5
	1.0	550.1 ± 37.9	5.1 ± 1.4	1.0 ± 0.3
	1.5	564.4 ± 26.8	6.8 ± 4.6	0.4 ± 0.6
	2.0	553.4 ± 65.8	7.0 ± 1.0	1.4 ± 0.1

**Table 2:** Appearance frequency of the amino acid of exposed protein parts in the purified intermediate. Fold change: values of the averaged protein intensity in the intermediate after hexane purification/averaged protein intensity in cell contents without hexane purification. Content ratio of amino acids (%): 100 × (each AA residue number of exposed protein parts/all AA residue numbers of the exposed protein parts). The highlight meant the higher occupied AA in the protein composition.

Fold-Change (B/A)	Protein Name		AA Residue- Number of Exposed Protein Parts	Composition of AA (%)																			
				A	C	D	E	F	G	H	I	K	L	M	N	P	Q	R	S	T	V	W	Y
83.62	Sulfatase N- terminal domain- containing protein		114	8.8	0.9	4.4	1.8	0.9	12.3	0.9	0.9	3.5	7.0	2.6	2.6	24.6	4.4	4.4	11.4	1.8	3.5	0.9	2.6
81.90	Repressor of RNA polymerase III transcription		38	15.8	0.0	7.9	0.0	5.3	10.5	2.6	2.6	5.3	7.9	0.0	0.0	15.8	2.6	0.0	5.3	5.3	5.3	0.0	7.9
81.51	UBC core domain-containing protein		163	6.1	3.1	5.5	6.1	2.5	7.4	1.8	4.9	3.1	8.0	3.7	3.7	8.6	3.1	7.4	9.2	3.1	7.4	2.5	3.1
65.07	DUF4347 domain-containing protein		226	7.5	0.0	0.4	5.3	0.0	7.1	0.9	0.4	1.8	0.9	0.4	0.0	45.1	0.0	1.3	14.2	3.5	4.4	0.0	6.2
35.74	BTB domain-containing protein		400	24.5	0.3	2.0	1.3	1.3	10.0	0.5	1.8	0.5	4.3	1.0	0.5	16.5	10.5	2.3	8.5	5.3	1.8	0.3	7.3
32.51	C3H1-type domain-containing protein	Arm A	171	15.8	0.6	0.6	2.9	0.6	19.9	1.8	1.2	1.8	4.7	5.3	7.6	4.1	4.1	2.3	17.5	6.4	2.9	0.0	0.0
		Arm B	227	26.9	0.0	0.4	0.4	0.4	15.0	4.8	1.8	0.0	7.9	4.8	1.8	4.4	13.7	1.3	5.7	2.2	5.3	0.0	2.6

## Data Availability

The data are contained within the article.

## Supplementary Materials

The following supporting information can be downloaded at https://www.mdpi.com/article/10.3390/bioengineering10080893/s1: Table S1: The frequently appearing proteins in purified intermediate.

## References

[1] Department of Economic and Social Affairs. https://sdgs.un.org/2030agenda.

[2] Ralston J., Cooper K., Powis J. (2021). Obesity, SDGs and ROOTS: A framework for impact. Curr. Obes. Rep..

[3] Plastic Production Worldwide 2021|Statista. https://www.statista.com/statistics/282732/global-production-of-plastics-since-1950/.

[4] Karaagac E., Jones M.P., Koch T., Archodoulaki V.M. (2021). Polypropylene contamination in post-consumer polyolefin waste: Characterisation, consequences and compatibilisation. Polymers.

[5] Center for International Environment Law. https://www.ciel.org/wp-content/uploads/2017/09/Fueling-Plastics-Fossils-Plastics-Petrochemical-Feedstocks.pdf..

[6] Adhikari D., Mukai M., Kubota K., Kai T., Kaneko N., Araki K.S., Kubo T. (2016). Degradation of bioplastics in soil and their degradation effects on environmental microorganisms. J. Agric. Chem. Environ..

[7] Lambert S., Wagner M. (2017). Environmental performance of bio-based and biodegradable plastics: The road ahead. Chem. Soc. Rev..

[8] Nakanishi A., Iritani K., Sakihama Y. (2020). Developing neo-bioplastics for the realization of carbon sustainable society. J. Nanotechnol. Nanomater..

[9] Eriksen M., Lebreton L.C.M., Carson H.S., Thiel M., Moore C.J., Borerro J.C., Galgani F., Ryan P.G., Reisser J. (2014). Plastic pollution in the world’s oceans: More than 5 trillion plastic pieces weighing over 250,000 tons afloat at sea. PLoS ONE.

[10] Ferreira-Filipe D.A., Paço A., Duarte A.C., Rocha-Santos T., Patrício Silva A.T. (2021). Are biobased plastics green alternatives?—A critical review. Int. J. Environ. Res. Public Health.

[11] Garavand F., Rouhi M., Razavi S.H., Cacciotti I., Mohammadi R. (2017). Improving the integrity of natural biopolymer films used in food packaging by crosslinking approach: A review. Int. J. Biol. Macromol..

[12] Hottle T.A., Bilec M.M., Landis A.E. (2013). Sustainability assessments of bio-based polymers. Polym. Degrad. Stab..

[13] Arraiza M.P., López J.V., Fernando A., Santamarta-Cerezal J.C., Gutiérrez L.E.H. Aerobic degradation of bioplastic materials. Environmental Security and Solid Waste Management, Proceedings of the 1st International Workshop on Environmental Security, Geological Hazards and Management, San Cristobal de La Laguna, Spain, 10–12 April 2013.

[14] Meixner K., Kovalcik A., Sykacek E., Gruber-Brunhumerabe M., Zeilinger W., Markl K., Haas C., Fritz I., Mundigler N., Stelzer F. (2018). Cyanobacteria biorefinery—Production of poly (3-hydroxybutyrate) with *Synechocystis salina* and utilization of residual biomass. J. Biotechnol..

[15] Nakanishi A., Iritani K., Sakihama Y., Ozawa N., Mochizuki A., Watanabe M. (2020). Construction of cell-plastics as neo-plastics consisted of cell-layer provided green alga *Chlamydomonas reinhardtii* covered by two-dimensional polymer. AMB Expr..

[16] Nakanishi A., Iritani K., Sakihama Y., Watanabe M. (2020). Investigation of the mechanical strength of cell-plastics fabricated using unicellular green algal cells and varying weight ratios of biodegradable polybutylene succinate. Int. J. Microbiol. Biotechnol..

[17] Nakanishi A., Iritani K., Sakihama Y., Watanabe M., Mochizuki A., Tsuruta A., Sakamoto S., Ota A. (2021). Fabrication and biodegradability of starch cell-plastics as recyclable resources. Appl. Sci..

[18] Iritani K., Nakanishi A., Ota A., Yamashita T. (2021). Fabrication of novel functional cell-plastic using polyvinyl alcohol: Effects of Nakanishi, A cross-linking structure and mixing ratio of components on the mechanical and thermal properties. Glob. Chall..

[19] Nakanishi A., Iritani K., Tsuruta A., Yamamoto N., Watanabe M., Ozawa N., Watanabe M., Zhang K., Tokudome A. (2022). Fabrication of cell-plastics composed only of unicellular green alga *Chlamydomonas reinhardtii* as a raw material. Appl. Microbiol. Biotechnol..

[20] Schreiber C., Schiedung H., Harrison L., Briese C., Ackermann B., Kant J., Schrey S.D., Hofmann D., Singh D., Ebenhöh O. (2018). Evaluating potential of green alga *Chlorella vulgaris* to accumulate phosphorus and to fertilize nutrient-poor soil substrates for crop plants. J. Appl. Phycol..

[21] Alvarez A.L., Weyers S.L., Goemann H.M., Peyton B.M., Gardner R.D. (2021). Microalgae, soil and plants: A critical review of microalgae as renewable resources for agriculture. Algal Res..

[22] Dementyeva P., Freudenberg R.A., Baier T., Rojek K., Wobbe L., Weisshaar B., Kruse O. (2021). A novel, robust and mating-competent *Chlamydomonas reinhardtii* strain with an enhanced transgene expression capacity for algal biotechnology. Biotechnol. Rep..

[23] Sathe S., Durand P.M. (2016). Cellular aggregation in *Chlamydomonas* (Chlorophyceae) is chimaeric and depends on traits like cell size and motility. Eur. J. Phycol..

[24] Williams J.G.K. (1988). Construction of specific mutations in photosystem II photosynthetic reaction center by genetic engineering methods in *Synechocystis* 6803. Meth. Enzymol..

[25] Ma N., Liu D., Liu Y., Sui G. (2015). Extraction and characterization of nanocellulose from *Xanthoceras sorbifolia* husks. Int. J. Nanosci. Nanotechnol..

[26] Dreywood R. (1946). Qualitative test for carbohydrate material. Ind. Eng. Chem. Anal. Ed..

[27] Morris D.L. (1948). Quantitative determination of carbohydrates with dreywood’s anthrone reagent. Science.

[28] Ho S.H., Nakanishi A., Ye X., Chang J.S., Chen C.Y., Hasunuma T., Kondo A. (2015). Dynamic metabolic profiling of the marine microalga *Chlamydomonas* sp. JSC4 and enhancing its oil production by optimizing light intensity. Biotechnol. Biofuels.

[29] Nakanishi A., Ho S.H., Kato Y., Yamasaki H., Chang J.S., Misawa N., Hirose Y., Minagawa J., Hasunuma T., Kondo A. (2017). Dynamic metabolic profiling together with transcription analysis reveals salinity-induced starch-to-lipid biosynthesis in alga *Chlamydomonas* sp. JSC4. Sci. Rep..

[30] Wang M., Yuan W., Jiang X., Jing Y., Wang Z. (2014). Disruption of microalgal cells using high-frequency focused ultrasound. Bioresour. Technol..

[31] Duan Z., Tan X., Guo J., Kahehu C.W., Yang H., Zheng X., Zhu F. (2017). Effects of biological and physical properties of microalgae on disruption induced by a low-frequency ultrasound. J. Appl. Phycol..

[32] Prewo K.M. (1986). Tension and flexural strength of silicon carbide fibre-reinforced glass ceramics. J. Mater. Sci..

[33] Callister W.D., Hayton J. (2007). Characteristics, applications, and processing of polymers. Materials Science and Engineering.

[34] Beer F.P., Johnston E.R., Dewolf J.T., Mazurek D.F. (2012). Mechanics of Materials.

[35] Ridwan R., Prabowo A.R., Muhayat N., Putranto T., Sohn J.M. (2020). Tensile analysis and assessment of carbon and alloy steels using FE approach as an idealization of material fractures under collision and grounding. Curved Layer Struct..

[36] Boey J.Y., Lee C.K., Tay G.S. (2022). Factors affecting mechanical properties of reinforced bioplastics. Polymers.

[37] Stauber E.J., Fink A., Markert C., Kruse O., Johanningmeier U., Hippler M. (2003). Proteomics of *Chlamydomonas reinhardtii* light-harvesting proteins. Eukaryot. Cell..

[38] Diniz M.C., Pacheco A.C.L., Farias K.M., Oliveira D.M.D. (2012). The eukaryotic flagellum makes the day: Novel and unforeseen roles uncovered after post-genomics and proteomics data. Curr. Protein Pept. Sci..

[39] Williamson M.P. (1994). The structure and function of proline-rich regions in proteins. Biochem. J..

[40] Fischetti V.A., Pancholi V., Schneewind O., Dunny G.M., Cleary P.P., McKay L.L. (1991). Common characteristics of the surface proteins from grampositive cocci. Genetics and Molecular Biology of STREPTOCOCCI, Lactococci and Enterococcil.

[41] Roditi I., Schwarz H., Pearson T.W., Beecroft R.P., Liu M.K., Richardson J.P., Buhring H.J., Pleiss J., Bulow R., Williams R.O. (1989). Procyclin gene expression and loss of the variant surface glycoprotein during differentiation of *Trypanosoma Brucei*. J. Cell Biol..

[42] Timoney J.F., Muktar M., Ding J., Dunny G.M., Cleary P.P., McKay L.L. (1991). M proteins of the equine group C streptococci. Genetics and Molecular Biology of Streptococci, Lactococci and Enterococci.

[43] Hedén L.O., Frithz E., Lindahl G. (1991). Molecular characterization of an IgA receptor from group B streptococci: Sequence of the gene, identification of a proline-rich region with unique structure and isolation of N-terminal fragments with IgA-binding capacity. Eur. J. Immunol..

[44] Pancholi V., Fischetti V.A. (1988). Isolation and characterization of the cell-associated region of group A streptococcal M6 protein. J. Bacteriol..

[45] Fahnestock S.R., Alexander P., Nagle J., Filpula D. (1986). Gene for an immunoglobulin-binding protein from a group G *Streptococcus*. J. Bacteriol..

[46] Li L.J., Dougan G., Novotny P., Charles I.G.P. (1991). 70 pertactin, an outer-membrane protein from *Bordetella parapertussis*: Cloning, nucleotide sequence and surface expression in *Escherichia coli*. Mol. Microbiol..

[47] Cronmiller E., Toor D., Shao N.C., Kariyawasam T., Wang M.H., Lee J.H. (2019). Cell wall integrity signaling regulates cell wall-related gene expression in *Chlamydomonas reinhardtii*. Sci. Rep..

[48] Mitaku S., Suzuki K., Odashima S., Ikuta K., Suwa M., Kukita F., Ishikawa M., Itoh H. (1995). Interaction stabilizing tertiary structure of bacteriorhodopsin studied by denaturation experiments. Proteins.

[49] Pekkala M., Hieta R., Kursula P., Kivirikko K.I., Wierenga R.K., Myllyharju J. (2003). Crystallization of the proline-rich-peptide binding domain of human type I collagen prolyl 4-hydroxylase. Acta. Crystallogr. D Biol. Crystallogr..

[50] Valenzuela C.D., Wagner W.L., Bennett R.D., Ysasi A.B., Belle J.M., Molter K., Straub B.K., Wang D., Chen Z., Ackermann M. (2017). Extracellular assembly of the elastin cable line element in the developing lung. Anat. Rec..

