# Supplementary material for: Identification of Cell-Attachment Factors Derived from Green Algal Cells Disrupted by Sonication in Fabrication of Cell Plastics
Source: Bioengineering (Basel). 2023 Jul 27;10(8):893. doi: 10.3390/bioengineering10080893 (PMC10451321; doi:10.3390/bioengineering10080893)
Supplement: Supplementary file 1 [file bioengineering-10-00893-s001.zip › bioengineering-2451707-supplementary.pdf]

Supplementary Materials

Table S1. Highly appeared proteins in purified intermediate

No.	Accession number	Fold-chage (B/A)	Protein name	EC number	Total number of amino acids	Location
1	A0A2K3DT53	83.62	Sulfatase domain-containing protein	-	688	-
2	A0A2K3DGS6	81.90	Repressor of RNA polymerase III transcription	-	235	Nucleus
3	A0A2K3E455	81.51	UBC core domain-containing protein	-	163	Nucleus
4	A0A2K3CSD3	65.07	DUF4347 domain-containing protein	-	587	-
5	A0A2K3DK02	41.65	Protein kinase domain-containing protein	2.7.1.171	1428	Actin filament
6	A0A2K3DX97	35.74	BTB domain-containing protein	-	801	Cytoplasm
7	A0A2K3DM36	32.81	Ferredoxin--NADP(+) reductase	-	83	-
8	A0A2K3DVE0	32.51	C3H1-type domain-containing protein	-	868	-
9	A0A2K3DZT1	31.27	Hydrolase_4 domain-containing protein	-	411	-
10	A0A2K3E279	30.89	SRCR domain-containing protein	-	1350	Membrane
11	A0A2K3E877	27.99	Guanylate cyclase domain-containing protein	2.7.7.65	2854	-
12	A0A2K3CPF2	27.96	PUM-HD domain-containing protein	-	1846	Membrane
13	A0A2K3DMR1	27.65	DUF952 domain-containing protein	-	141	-
14	A0A2K3CPT2	26.21	Non-specific serine/threonine protein kinase	2.7.11.1	1590	Membrane
15	A0A2K3E5V4	26.11	No exine formation 1	-	1200	Membrane
16	A0A2K3D6J3	25.87	Rab3 GTPase-activating protein catalytic subunit	-	395	-
17	A0A2K3DYL9	25.51	ANAPC4_WD40 domain-containing protein	-	659	Nucleus
18	A8J3A7	25.48	Small ubiquitin-related modifier	-	97	Nucleus
19	A0A2K3E6F6	22.68	Histone acetyltransferase	2.3.1.48	1160	Nucleus
20	A8HY76	21.14	Hydroxyethylthiazole kinase	2.7.1.50	277	–
21	A0A2K3D1J8	20.01	RRM domain-containing protein	-	734	Commitment complex, nuclear speck, U2-type prespilceosome , U2AF complex
22	A0A2K3D8H7	19.32	Uncharacterized protein	-	329	-
23	A0A2K3CTV5	19.31	Snurportin-1	-	598	Nucleus, Cytoplasm
24	A8JBP0	18.28	Serine/threonine-protein phosphatase	3.1.3.16	314	Cytosol
25	A0A2K3D0Q6	17.95	DIOX_N domain-containing protein	-	451	-
26	A0A2K3DWW3	17.14	CLP1_P domain-containing protein	-	735	Nucleus
27	A8HXV4	16.98	Uncharacterized protein	-	681	Cytosol, colgi membrane, Ric1-Rgp1 guanyl-nucleotide exchange factor complex
28	A0A2K3DQT5	16.84	Fibronectin type-III domain-containing protein	-	486	-
29	A0A2K3DKZ4	16.63	PseudoU_synth_2 domain-containing protein	-	926	-
30	A0A2K3CQT5	16.19	Protein kinase domain-containing protein	2.7.1.171	4006	Membrane
31	A0A2K3DWR3	15.82	Phosphoglycerate mutase family protein	5.4.2.4	304	-
32	A0A2K3DQ81	15.62	Trafficking protein particle complex subunit	-	126	Endoplasmic reticulum, Golgi apparatus, cis-Golgi network
33	A0A2K3E0F7	15.20	DIOX_N domain-containing protein	-	321	-
34	A0A0U2Q5E3	14.57	NER endonuclease	-	2463	Nucleus
35	A8IZN1	14.57	Uncharacterized protein	-	867	Cell periphery, endoplasmic reticulum, membrane
36	A0A2K3CXS9	14.46	Uncharacterized protein	-	138	-
37	A0A2K3E015	14.39	TFIIS central domain-containing protein	-	1373	Nucleus
38	A8JHA7	14.21	Uncharacterized protein	-	145	-
39	A0A2K3DDC7	14.04	SpoU_methylase domain-containing protein	-	304	-
40	A8I202	13.60	Uncharacterized protein	-	269	Endomembrane system, SNARE complex
41	A0A2K3CTM8	13.57	CYTH domain-containing protein	-	275	-
42	A0A2K3DU48	13.50	MMS1_N domain-containing protein	-	427	-
43	A0A2K3E7Z1	13.06	RanBD1 domain-containing protein	-	572	-
44	A0A2K3DW99	12.68	HECT-type E3 ubiquitin transferase	2.3.2.26	1291	-
45	A8HQM9	12.59	Uncharacterized protein	-	353	Endoplasmic reticulum
46	A0A2K3D5Q4	11.54	Lactamase_B domain-containing protein	-	272	-
47	A0A2K3D7I1	10.32	Cytochrome P450	1.6.2.4	561	Membrane
48	A0A2K3D7M1	10.03	ANK_REP_REGION domain-containing protein	-	557	Membrane
49	A0A2K3E0X7	10.02	Sulfatase domain-containing protein	-	1036	-

Forty-nine proteins grouped in Cluster No. 2, which were 10 times larger the protein intensities of the purified intermediate divided by those of the cell-content, were displayed. Fold change: Values of averaged protein-intensity in intermediate after hexane-purification / averaged protein-intensity in cell-contents without hexane-purification.
